# Systematic optimization and evaluation of a Dutch sexual health intervention: Role model stories for chlamydia prevention, testing, and treatment

**DOI:** 10.1177/20552076241308447

**Published:** 2025-01-23

**Authors:** Gido Metz, Rosa RLC Thielmann, Hanneke Roosjen, Sarah E. Stutterheim, Rik Crutzen

**Affiliations:** 1 Department of Health Promotion, 5211 Maastricht University/CAPHRI, Maastricht, The Netherlands; 2 26164 Soa Aids Nederland, Amsterdam, The Netherlands

**Keywords:** Behavior change, sexual health, mixed methods, eHealth, public health, evaluation, think-aloud, web analytics, role model stories

## Abstract

**Background:**

The rapidly evolving nature of eHealth necessitates regular optimization and subsequent evaluation. Within the Dutch sexual health intervention Sense.info, we utilized a mixed-methods cyclic evaluation process to assess and optimize the potential impact of the chlamydia page. This paper reports on the page's optimization through the development of role model stories for chlamydia prevention and the subsequent evaluation of these stories.

**Method:**

The experiences of 10 young individuals served as the basis of role model stories using the behavior change principle modeling based on social cognitive theory. These stories aimed to motivate young individuals to undergo sexually transmitted infection testing, use condoms, and notify sexual partners. Once the stories were posted online, we tracked use data between July and September 2022 and investigated end-user perspectives through a think-aloud study combined with semistructured interviews (*N *= 20, *M*_age _= 19.7, SD_age _= 2.65). Template analyses were used for the analysis of the think-aloud study.

**Results:**

Use data revealed that all stories were accessed by website visitors, yet other page elements on the chlamydia page interacted with more. The exploration of end-user perspectives indicated a positive impact of the personal stories on normalization, self-efficacy, and skills related to chlamydia preventive behaviors. Mixed results were found regarding some conditions for the effectiveness of the behavior change principle modeling.

**Discussion and conclusion:**

This study provided valuable insights into the cyclic evaluation process for evaluating and optimizing web-based public health interventions, as well as the potential impact of role model stories on sexual health prevention. Also, aspects of the stories that could be optimized in future optimization rounds were identified. Overall, this research contributes to enhancing the impact of eHealth interventions through iterative evaluation and optimization processes.

Regular optimization of eHealth is essential given the rapid advancements in technology and content. Each of these optimizations requires evaluation. Although randomized controlled trials (RCTs) remain the gold standard when it comes to rigor, they have drawbacks when it comes to eHealth evaluation. Conducting an RCT requires a process involving the selection of clear primary outcomes, careful randomization, and no changes to the intervention content or its delivery method.^1^ This process not only misaligns with the fast-paced developments in technology and content but also with web-based interventions typically being accessible to a wide range of users over an extended period, making randomization difficult.^2,3^ Furthermore, web-based public health interventions are often described as “complex interventions,” meaning they typically involve multiple interacting elements, have multiple target behaviors and outcomes, and incorporate the ability to tailor intervention content.^4^ Over the years, alternative research methods have become increasingly viable options.^5,6^ For example, in the Dutch and British intervention evaluation and recognition frameworks, qualitative and mixed research methods are now also deemed valuable, besides RCTs, in ascertaining intervention effectiveness.^5,7^

Following this recognition of the utility of mixed-methods research, we developed and utilized a mixed-methods approach comprising several phases to evaluate and optimize the chlamydia page of the Dutch sexual health intervention Sense.info. This website is the primary platform for accessing information and services related to sexual and reproductive health for individuals aged 12–25 in the Netherlands. Sense.info encompasses six main aims, which are familiarizing oneself with one's body, developing a sexual identity, experiencing sexual pleasure, and preventing boundary crossing, sexually transmitted infections (STIs), and unwanted pregnancies.

## Cyclic evaluation process

**Figure 1.**
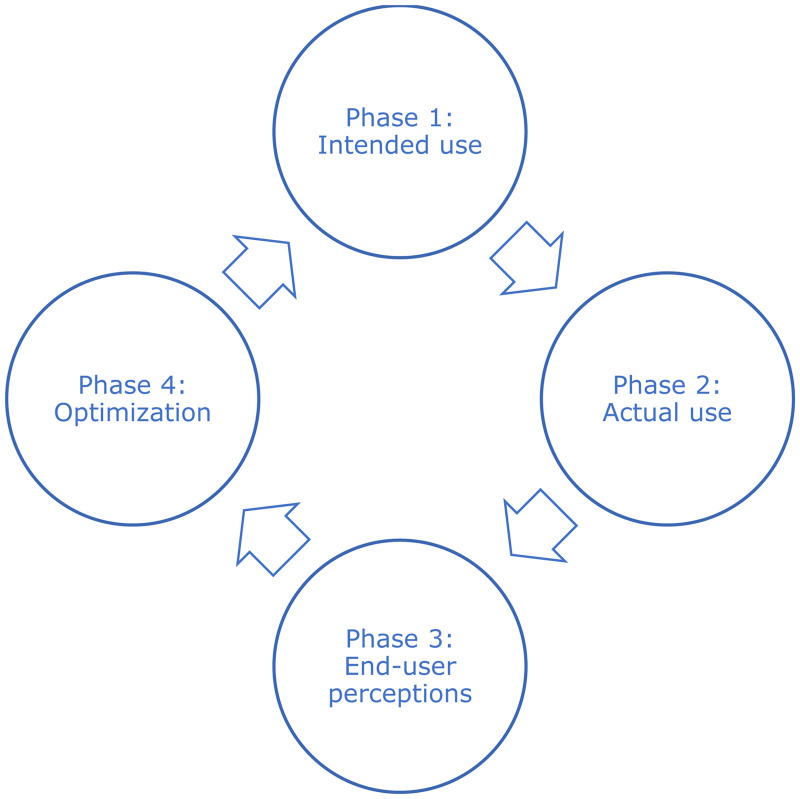
Cyclic evaluation process.

The mixed-methods approach used, the cyclic evaluation process (CEP), brings together a number of methods in four phases (see Figure 1). The first phase involves a theoretical analysis of intended use, which sheds light on the intervention developers’ vision regarding intervention use and the resulting behavior change. This phase holds particular significance due to the complexity of eHealth interventions and the concerns raised about inadequate reporting of interventions, hindering analyses, replications, or reuse.^8–11^ Acyclic behavior change diagrams (ABCD), serving as an operational tool to visualize the active components of behavior change interventions,^12^ are employed in this phase. An ABCD comprises multiple strings of seven boxes, depicting the causal–structural chains underlying an intervention. The seven boxes visualize (a) the behavior change principles (BCP), taking into account (b) the conditions for effectiveness, as applied in (c) a specific application, addressing (d) subdeterminants and (e) determinants, relevant to changing (f) the necessary subbehaviors and, ultimately, the (g) target behavior.

In the second phase, discrepancies between intended use and actual use are examined using web analytics. Following these two phases, assumptions can be made regarding the potential impact. In the third phase, these assumptions are further explored through a combination of the think-aloud method and semistructured interviews. Subsequently, optimization is carried out in the fourth phase, building upon the insights gained from the three previous phases. The fourth phase also involves analyzing the intended use of the newly developed or optimized elements, thus returning to the first phase.

The CEP is based on four key characteristics, the first of which is iteration. Web-based interventions offer the advantage of easy and rapid adaptation, along with continuous monitoring of end-user behavior, in contrast to RCT procedures with fixed intervention protocols that remain unchanged throughout the study period.^1,2,13^ An iterative approach has the potential to enhance effectiveness systematically and incrementally.^14–16^ Second, several forms of triangulation are employed within the CEP, resulting in what Denzin describes as “multiple triangulation.”^17^ Data triangulation, which entails the amalgamation of data collected at different times, places, settings, or from different individuals, is realized in the CEP through the iterative analysis of use data and exploration of end-user perspectives. Investigator triangulation, or the involvement of multiple observers, interviewers, coders, or data analysts in the study, is implemented in at least this study, as multiple researchers with different expertise participate throughout all phases. Within-method triangulation, or the utilization of at least two data-collection procedures within the same design approach (i.e., quantitative or qualitative research), is applied through the combination of the qualitative think-aloud and interview methods in phase 2. Between- or across-method triangulation, the application of both qualitative and quantitative methodology is achieved by combining a theoretical analysis of intended use and a quantitative analysis of actual use, followed by a qualitative exploration of end-user perspectives. Theoretical triangulation, or the use of several theories and hypotheses when examining a phenomenon, is applied throughout all phases of the process, allowing for the integration of various theories. For instance, in the first phase, when analyzing the intervention elements and their potential contributions to behavior change, taxonomies from frameworks such as intervention mapping,^9,18^ the behavior change wheel,^19,20^ and other intervention planning frameworks can be employed to categorize the intervention elements. These taxonomies are grounded in multiple theories. Similarly, during the development of new elements in the optimization phase, several BCPs rooted in different theories can be amalgamated within the new elements.

The third key characteristic pertains to the ease and speed with which the CEP can be deployed. Methods used within the CEP are intended to be user-friendly and swift for web-based public health intervention developers. Given that web analytics and think-aloud studies have been familiar concepts in the realm of (web-based public health intervention) development and usability research and practice,^21,22^ the CEP is expected to serve as a relatively straightforward approach for most intervention developers. The fourth and final characteristic of the CEP is meaningful community engagement, facilitated by the qualitative methods involved.^23^ From a utilitarian and functional perspective, involving the community (e.g., intervention users and intermediaries) is expected to enhance intervention effectiveness.^24^ Viewed through a social justice lens, community engagement can support and empower individuals and communities, particularly when aligned with key values such as dignity and nonothering.^23–27^ Community engagement can be seen as a continuum that moves from outreach efforts to consultation, followed by community involvement, then collaboration, and, finally, shared leadership and decision-making.^26^ In the context of the CEP, the community of (representatives of the) end-users is involved both indirectly and directly. Indirect involvement occurs through the analysis of their use data in phase 2, leading to assumptions about the impact of the intervention on the community. Direct involvement takes place when end-user perceptions are explored in phase 3. These perspectives can guide the optimization in phase 4, where input from other end-users can once again be sought.

## CEP in the context of Sense.info

**Figure 2.**
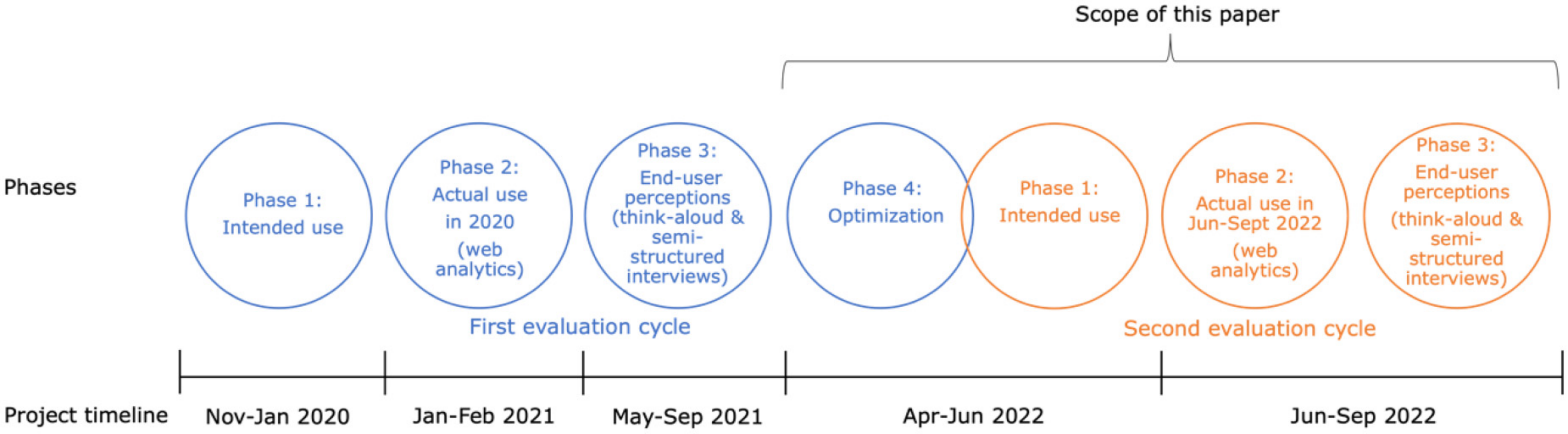
The phases of the cyclic evaluation process conducted in the Sense.info project. Phases 1 to 3 of the first evaluation cycle (in blue) have been reported on in previous papers.^28,29^ *Note:* Phase 4 of the first evaluation cycle and phase 1 of the second evaluation cycle (in orange) are intertwined due to the necessity of considering intended use while developing new elements in the optimization phase.

The cyclic nature of the CEP allows for subsequent rounds of evaluation and optimization, as demonstrated in the Sense.info project (Figure 2). This paper specifically focuses on the optimization and evaluation that followed the initial evaluation cycle. For context, the main findings of the first evaluation round will be described in the next paragraph, and a more comprehensive description of this evaluation can be found elsewhere.^28,29^

The analysis of intended use (phase 1) of the Sense.info chlamydia page revealed its objective of providing knowledge about the symptoms and consequences of chlamydia. Additionally, it aimed to motivate young people to undergo STI testing, use condoms, and notify their sexual partners in case of potential chlamydia transmission. The analysis of actual use (phase 2) indicated a relatively low percentage of page transfers from the chlamydia page to the page containing information on STI testing.^28^ During the exploration of end-user perspectives (phase 3), participants suggested, among other things, the inclusion of personal stories on the page to motivate STI testing.^29^ Consequently, in the optimization phase (phase 4) on which the current paper reports, we created role model stories about chlamydia. We followed a participatory approach, collaborating with young individuals who had experienced chlamydia to develop the stories. The main objectives of these stories were to normalize chlamydia and encourage condom use, STI testing, STI treatment, and partner notification.

### Role model stories

Role model stories, also referred to as narratives or testimonials, have been shown to successfully impact various health behaviors.^30^ Over time, several theoretical approaches have been used for the development of these stories and to explain their success.^31^ A recent scoping review revealed common success factors to be the use of first-person storytelling, fostering similarity and identification with the protagonist, and gathering input from the target audience to tailor the narratives effectively.^32^ Other factors explaining the effect of narratives include that they reduce reactance and counterarguing in recipients, increase perceived vulnerability and self-efficacy, and change perceived norms or outcome expectancies.^33^ Over the years, research on social cognitive theory has indeed emphasized the influential role of self-efficacy in individuals’ adoption and maintenance of health behaviors.^34–36^ One of the ways to increase self-efficacy is through modeling, meaning that observing a similar other succeed by perseverant effort raises observers’ beliefs in their own abilities.^35,37^ The present study aims to utilize modeling as a BCP underlying the role model stories. A narrative-based approach was well-aligned with Sense.info's existing communication strategies, as the website has long had a section where young people can share their personal experiences. However, not many personal experiences on the topic of STIs have been shared in the past. Also, in the past, the Sense.info personal narratives were not necessarily written in a way that optimally incorporated BCPs and their conditions for effectiveness. Therefore, in this study, our primary objective was to strike a balance between the personal experiences of young individuals serving as input for the stories and the conditions for the effectiveness of the BCP modeling. In addition, the overall aim of the paper is to illustrate the use of the CEP as applied to the development and evaluation of role model stories for the Sense.info website.

## Methods and results

The study was conducted in the Netherlands. This section outlines the methods and results of all phases of this mixed-methods study. These include the development of role model stories, analysis of use data, and the exploration of user perceptions through a think-aloud study combined with semistructured interviews.

### Optimization and intended use phase: developing role model stories

#### Method

The role model stories addressing chlamydia prevention, screening, and treatment behaviors were founded on the experiences of 10 young individuals, shared during interviews. We chose 10 role model stories in total as we expected that this number of stories would lead to enough variation in terms of different target behaviors (i.e., STI testing, notifying partners, using condoms), strategies (e.g., testing with a self-test kit or at the Sense consultation hours at municipal health centers specifically focusing on the young individuals in the Sense.info target group), and demographic background of the interviewees (e.g., gender identity, working or being enrolled in some form of education).

*Inclusion criteria.* Interviewees were required to fall within the age range of 16 and 25, aligning with Sense.info's target demographic. This approach aimed to have the recipient identify with the story's protagonist. As mentioned in the previous paragraph, we also sought variation in both the specific story contents and the background characteristics of the protagonists, expecting to foster identification.

*Sampling and recruitment.* The 10 interviewees were recruited through a banner on Sense.info that read: “Share your story about chlamydia and receive €25. We are seeking young people who wish to share their stories anonymously. Reward: €25 gift card.” The banner led to an information page with a link to an intake questionnaire, requesting the interviewees’ first name (for verification purposes during the phone call), age, gender identity, email address, and phone number. It also provided the option to write down (parts of) the story, though this was not mandatory, as our goal was to be inclusive of individuals who might not have felt inclined or capable of doing so. The questionnaire was completed by 46 individuals, of which 13 did not write down their stories. We ensured to also call individuals who did not write down their stories, resulting in a sample of five young individuals who had written down their stories and five who had not.

**Figure 3.**
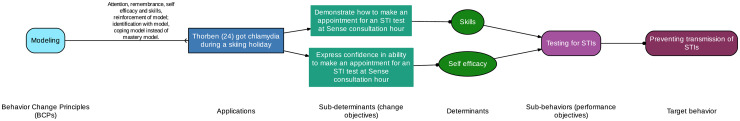
Acyclic behavior change diagram (ABCD) of one of the personal stories.

*Procedure.* Interviews were held via phone in April 2022. The researcher followed an interview protocol (Appendix A) that was based on the ABCD which we designed to visualize the determinants (i.e., skills and self-efficacy) and subdeterminants (e.g., demonstrate how to make an appointment for an STI test at Sense consultation hour) essential for targeting the subbehaviors (e.g., testing for STs, notifying sexual partners) and the overarching target behavior (preventing transmission of STIs), alongside the conditions for effectiveness of the BCP modeling (a) the reader's attention must be drawn to the story, (b) they must be able to remember the main message, and (c) they must be able to identify with the protagonist; (d) the role model story should target self-efficacy and skills and (e) include reinforcement of the behavior, and (f) the protagonist should be a coping model rather than a mastery model) (Figure 3).

After the interview, the interviewee and researcher agreed upon a fictitious name for the protagonist, and a digital gift card was sent to the interviewee by e-mail. The researcher wrote down the story taking into account the conditions for effectiveness for modeling and then sent it to a medical doctor at Soa Aids Nederland for medical accuracy verification and to a text writer to align it with other Sense.info stories in tone and layout. The research team then checked if the stories still met the conditions for effectiveness. The interviewee had the chance to read and propose changes to the story. Some provided suggestions, and adjustments were made accordingly. Subsequently, all interviewees provided written informed consent for their stories to be published on Sense.info.

#### Results

The 10 interviews were developed into personal stories with particular attention to the target behavior, subbehaviors, determinants, and conditions for effectiveness of the developed ABCDs. Considering the conditions for effectiveness, we included aspects of the young individuals’ personal background and everyday life in the stories to help recipients identify with the characters and be drawn to the narratives due to their personal relevance. This personal relevance was intended to aid in remembering the main message. For instance, one story began with “last summer, I met a cute guy at my holiday job. We weren’t really in a relationship, but we did have occasional sex.” Another story started with “I broke up with my boyfriend a while back. To forget him, I went online to look for a one-night stand.” To address reinforcement, coping models, skills, and self-efficacy, we asked the interviewees about their initial feelings upon suspecting chlamydia, the steps they took for STI testing, seeking treatment, and partner notification. We also inquired about the initial challenges they encountered, the subsequent ease they experienced during these steps, and the positive outcomes. For example, one interviewee described their experience with an STI test, from their initial apprehension to the actions during the appointment and the relief afterward:

How I got tested? I made an appointment at the sexual health center. First, you have to complete a checklist online. Then you have a call with a nurse. That was all quite easy. We walked through the checklist together. After two days, I could pass by the center.

I expected it to be awkward, but that was not the case at all. It was all very professional, and I felt at ease. I got information about chlamydia and a phone number in case I’d have more questions. I remember walking out relieved.

Links to two of the personal stories were placed on the chlamydia page along with links to the general “Personal stories” page, containing all other stories on chlamydia. The names of the respective stories can be found in Table 1. All role model stories are available in the Open Science Framework Repository at https://doi.org/10.17605/osf.io/bcr48.

**Table 1.** Outgoing traffic from the Sense.info chlamydia page, July–September 2022.

Web pages	Count (%)
Types of STIs	1277 (32.42%)
Others	1058 (26.86%)
Advice chat	736 (18.68%)
Gonorrhea	224 (5.69%)
Nearby addresses	223 (5.66%)
Personal stories	188 (4.77%)
STI test	175 (4.44%)
10 tips to notify someone about STIs (www.soaaids.nl)	25 (0.63%)
Chlamydia (website www.thuisarts.nl)	15 (0.38%)
Chlamydia (website www.soaaids.nl)	11 (0.28%)
Text-to-speech reader	7 (0.18%)
Total	3939 (100%)

### Analysis of actual use phase: web analytics

#### Method

Following the placement of stories on the Sense.info chlamydia page, the analysis of actual use (phase 2) started. From July to September 2022, web analytics were monitored using Matomo, a secure open-source web analytics platform that adheres to current standards of data protection in accordance with the European General Data Protection Regulation.^38,39^ To ensure the accuracy and relevance of the data, we focused solely on visitors located in the Netherlands. This decision was made as visitors from abroad would have been able to read the stories but would not have had the opportunity to schedule an appointment during the Sense consultation hours.

*Results.* Table 2 reveals that all stories were accessed during the period from July to September 2022. The most frequently accessed story (about “Madelief (19),” who “felt guilty for having chlamydia”), was one of the stories with a direct link to the chlamydia page. However, compared to all other options to leave the chlamydia page (Table 1), this story did not rank very high; in its web analysis report, Matomo categorized data for this story and the other with a direct link on the chlamydia page as “Others.” This might indicate that end-users are less interested in the personal stories than in other page elements, such as the elements directing to the page “Types of STIs” or to the advice chat which offers tailored advice regarding the need for STI testing. The general “Personal stories” page, which contains all personal stories, was accessed 188 times from the chlamydia page. On average, visitors spent around one minute on the story pages. Bounce rates (i.e., the percentage of sessions during which visitors left the website after viewing only one specific page) ranged from 0% to 47% and exit rates (i.e., the percentage of sessions that finished on a page) from 13% to 49%. Based on the page views, we assume that the stories, to some extent, meet a need. However, further investigation is necessary, also regarding other metrics such as the low number of page transfers from the chlamydia page, time spent on the story pages, and bounce and exit rates.

**Table 2.** Use data personal stories July–September 2022.

Personal stories	Page views	Unique page views	Bounce rate	Average time spent on page	Exit rate
Angelino (25) got a severe form of chlamydia	100	82	0%	00:01:07	15%
Anne (21) got chlamydia twice	260	172	35%	00:00:46	63%
Dorothy (21) took a test twice*	162	133	0%	00:01:10	14%
Isabel (23) sometimes has a threesome	234	168	33%	00:00:48	49%
Joep (18) has sex with several girls	259	199	47%	00:00:54	41%
Madelief (19) felt guilty for having chlamydia*	325	277	45%	00:01:00	17%
Merel (19) now always uses condoms	170	157	0%	00:00:59	13%
Mila (23) got chlamydia from her boyfriend’s ex	76	49	16%	00:01:03	45%
Niels (23) did an STI test	106	79	0%	00:01:23	18%
Thorben (24) got chlamydia during a skiing holiday	56	46	0%	00:01:00	17%

*Note:* The story titles marked with a * are those with a direct link on the chlamydia page.

### Exploring end-user perceptions: think-aloud study and semistructured interviews

From June to September 2022, the assumed impact of the stories was further studied by exploring end-user perceptions with a think-aloud study combined with semistructured interviews (phase 3). The think-aloud method, in which participants navigate the intervention and view its content and concurrently verbalize their thoughts, is useful in understanding cognitive processes and emotional reactions.^40–42^ The Consolidated Criteria for Reporting Qualitative Research checklist was used in the design and reporting of the think-aloud study.^43^

#### Method

*Inclusion criteria.* Having had sexual interactions was a first inclusion criterion as it was expected that the chlamydia page was mostly visited by sexually active people and was also most relevant for this group. As a second criterion, we decided to include individuals aged 16 to 25. This age range aligns with reports about the age at which young people in the Netherlands typically begin having sex^44^ and is also consistent with a portion of the Sense.info target group.

*Recruitment.* Participants were recruited through a banner on Sense.info that appeared after visitors spent more than a minute on a page or when they navigated to another page on the website. Clicking on the banner led to a page with general information about the study and a link to a short intake questionnaire (created in *FormDesk*) asking for sociodemographic variables (age, being sexually active, gender identity, sexual orientation, being enrolled in a form of education or not, educational level, place of residence, birth country, birth country of parents). When the answers met the inclusion criteria, contact details (email address and phone number) were requested. Contact details were deleted after the completion of the study. If inclusion criteria were not met, people were automatically forwarded to a “thank you” page. The online intake questionnaire was accessible between June 10, 2022 and September 16, 2022, and was filled out 144 times. Of those, 24 entries did not meet the inclusion criteria, which resulted in a group of 120 eligible potential participants.

Based on the answers given in the intake questionnaire, purposive sampling was used.^45^ In the first weeks of recruitment, all eligible participants received a phone call from the researcher, giving them more information about the study procedure and asking them if they were still interested in participating. To ensure a diverse sample, efforts were made to select participants with various sociodemographic characteristics, taking into account the profiles of the initial participants.

If participants expressed interest in participating during the phone call, an appointment for the think-aloud interview was scheduled. The researcher emailed the participant a link to a Zoom meeting and an information letter containing all the information that had also been shared during the phone call with the participant. During the phone call, participants were asked which device they most often used to visit Sense.info, and they were asked to also use that device during this study. Eleven participants took part in the study from their laptops, the remaining nine from their mobile phones. The final sample consisted of 20 participants. Participant characteristics can be found in Tables 3 and 4.

**Table 3.** Participant characteristics.

	*n*
Gender identity	
Woman	10
Man	8
Man and nonbinary	1
Nonbinary	1
Age (*M* = 19.7, *SD* = 2.65)	
16	3
17	3
18	3
19	0
20	1
21	4
22	2
23	3
24	1
Sexual partners	
Woman having sex with women and men	2
Woman having sex with men	6
Woman having sex with women	1
Woman—pansexual	1
Man having sex with women and men	2
Man having sex with women	3
Man having sex with men	3
Nonbinary having sex with women and men	2
Place of birth of the participant	
The Netherlands	15
Iran	1
Suriname	1
Brazil	1
Syria	1
Kurdistan	1
Place of birth of one or both parents of the participant	
Netherlands	14
Iran	1
Surinam	1
Brazil	1
Syria	1
Kurdistan	1
Greece	1

**Table 4.** Educational level of the participants.

	Current form of education of participants currently enrolled in education	Last completed form of education of participants not enrolled in education anymore
Primary education		1
Lower secondary education	2	1
Upper secondary education	4	
Postsecondary nontertiary education	7	3
Tertiary education	1	1
Total	14	6

*Procedure.* Interviews were conducted from June to September 2022 until three consecutive interviews did not yield new relevant knowledge.^46^ The interviews were carried out through video calls with a professional Zoom account. GM (a PhD candidate in health promotion, identifying as a man, with an educational background in social psychology and law (MSc, LLM), and trained in qualitative research methods) conducted the interviews. No one other than the participant and the researcher was present during the interview. There was no prior relationship with the participants, other than a telephone call to explain the study and to arrange a time and date for the think-aloud study. Participants were aware that GM was a researcher at the Department of Health Promotion at Maastricht University and that the study was conducted independently of Soa Aids Nederland (one of the developers of Sense.info). Participants were informed beforehand that Maastricht University and Soa Aids Nederland shared the mutual goal of evaluating and optimizing Sense.info. No other details about the interviewer were disclosed to the participants. The duration of the appointments was approximately one and a half hour. In the first few minutes of the scheduled appointment, the researcher explained the study and the procedure following the participant’s letter. The participant then gave informed consent via a digital form (using *FormDesk*) after which the participant shared their screen and a practice think-aloud session started. The participant was asked to view the homepage of the website of the Trimbos Institute (a website dealing with health topics but not containing information about sexual health in general or chlamydia specifically) and perform a search task (looking up Trimbos Institute's phone number), all while thinking aloud.

After practicing and assuring that the participant understood the procedure, the think-aloud procedure for the Sense.info website started. Screen and audio were recorded with *QuickTime*. Participants were asked to navigate the homepage of Sense.info while thinking aloud. When they indicated having seen everything on the homepage, the researcher asked them to search for the chlamydia page and interact with this page the way they would do when they were alone while thinking aloud. The researcher emphasized that they were free to read texts, watch videos, and click on links to different pages (i.e., there were no limitations in terms of what content to choose and how long to use it). If participants were silent for over 10 s, the researcher used the prompt “feel free to think aloud.”

When participants indicated that they had seen enough, the second part commenced: an in-depth semistructured interview using an interview protocol (Appendix B). Regarding the personal stories, questions were asked focusing on the conditions for effectiveness of modeling (e.g., “to which degree could you identify with the young individual in the story?”) and on the specific determinants that were targeted (e.g., for self-efficacy: “to what degree do you feel you would manage to take an STI test after reading this story?”). Participants were reimbursed with a €25 gift card at the end of the interview.

*Data processing and analysis.* Think-aloud sessions and semistructured interviews were transcribed verbatim and then pseudonymized, with participant names replaced by unique participant numbers. Additionally, any other potentially revealing information was anonymized, such as instances where participants gave the exact location of their STI testing center, which could indicate their place of residence. Transcripts were subsequently sent to participants for confirmation of accuracy and approval for further use. Template analysis^47^ was used to analyze the transcripts in *Atlas.ti 9.* Initially, the coders (GM and RRLCT) familiarized themselves with the data by reading through it. They then proceeded to perform preliminary coding on a subset of the data, leading to an initial coding template consisting of both inductive themes and tentative a priori themes related to the targeted determinants, subbehaviors, and conditions for effectiveness of the BCP modeling. The initial template was independently applied to 10% of the data by both coders. They then discussed the coding; no major changes were made to the coding template. This version of the template was then again independently applied to another 10% of the dataset based on which intercoder-agreement was calculated using Krippendorff's alpha.^48,49^ This alpha was .82, indicating a high level of agreement among the raters.

#### Results

*Normalization and behavioral intentions.* Many participants expressed that the personal stories contributed to the normalization of chlamydia and preventive behaviors. In addition, some participants indicated that the stories also led to behavioral intentions to, for example, have sex with a condom or to take an STI test.

I think it is brave that this girl also shared her story. Because I couldn’t do that, but I do think it's nice to learn from other experiences [Yes, and can you tell me what you like about that?] Yes, then you get the feeling that you are not the only one and that you don't have to feel weird, like you are not sick, but you do have something that maybe has to be examined and that you have to be helped by people who have knowledge about it, and if you get the right treatment, then it should be okay. (P15)

*Skills and self-efficacy.* Regarding the target behavior “testing” in particular, some of the participants noted that the personal stories had a positive impact on self-efficacy.

She was relieved after the test and it was done very professionally. That's good to hear, because it gives me the confidence that if I have to do it myself sometime I can just go easily. (P08)

For skills regarding testing as well, some participants indicated that the personal stories had a positive impact, specifically referring to being able to follow the same steps as the protagonist.

It's just easier to understand what it all is if you read it as a person's experience, rather than just pieces of information. By following a person's experience, you can grasp the step-by-step process they followed, which can then provide you with information on how to take care of it yourself. (…) For example, the checklist, and then testing if necessary, and calling [sexual partners] if necessary. (P08)

*Perceived relevance of the stories.* Some participants mentioned spontaneously that they liked the presence of personal stories on the chlamydia page. Some other participants, however, noted that they were not entirely satisfied with the positioning of the links to the personal stories and indicated that they found the stories less interesting or relevant than the other information present on the chlamydia page.

The format, (…) that you put the symptoms at the top, so you can immediately say ‘okay, I think I might have this’ and then you could have that [digital] STI check possibly right after that, and only after that I would have the other information and stories of others, I personally find that less interesting. I would rather look up such a website to be able to find out if I have it or not. (P20)

*Identification.* As stated before, being able to identify with the role model in the story is one of the conditions for effectiveness of the BCP modeling, and this also seemed important to a few of the participants.

[Researcher: do you have any wishes in terms of the person telling the story?] Yes, diverse is always better. For example, this is a 19 year old girl. There might be a lot of 19 year old girls who say ‘this really speaks to me’, but a 24 year old man could also look this up and then ‘19 year old Madelief’ is maybe not the most representative person. (P05)

Identification of the participant with the role model depended on whether the participant was dealing with chlamydia at the time of the interview or had dealt with chlamydia in the past and whether they had multiple sexual partners or engaged in behavior such as condomless sex. A few participants were not able to identify with the protagonists.

[Researcher: To what extent can you identify with this young individual?] In a way, yes. For example, I too have taken some risks, and that he takes his own responsibility for it, that he doesn’t try to blame it on his partner. I have also done some stupid things but I have done that myself as well, so you have to take responsibility for that too. (P09)

[the role model having sex with] four to five girls in the last couple of weeks, I thought that was… well, a lot… yes. (…) Earlier on, the story also mentioned ‘friends with benefits’, then I assume you have one person, and that that is enough, but well. That's how I perceive it anyway. Although I have no experience with that, but well. [Researcher: to what extent then can you recognize yourself in this young person, in Joep?] That's a very good question. Not very much, but that's because I don’t have very much experience in this area so to speak. Or well, not with different people. So, no, not really. (P07)

*Coping models and reinforcement.* Other conditions of modeling are that protagonists should portray a progression from initial struggle in executing the target behavior to eventual success (i.e., a coping model) rather than exhibiting immediate mastery of the behavior (i.e., a mastery model) and that there should be positive reinforcement of the target behavior. A few participants felt that the outcomes of the personal stories were all positive and therefore questioned the authenticity of the stories.

It seems to be a made-up story, with the ‘no one will get mad at you and you won’t feel guilty’. (…) I can imagine that some people don’t respond well. Maybe also tell something about that. Because maybe in some cases people don’t respond well and then you might think: ‘Shouldn’t everyone have responded well?’. (P10)

## Discussion

This paper reports on the development and subsequent mixed-methods evaluation of role model stories for chlamydia prevention. The 10 stories, developed in close collaboration with young people, were evaluated using web analytics and a think-aloud study combined with semistructured interviews. From July to September 2022, all stories were accessed, with visitors spending approximately 1 min on the pages. Bounce and exit rates were relatively low. An analysis of how visitors left the chlamydia page showed that the two personal stories with a direct link on the chlamydia page were clicked on relatively infrequently. In the think-aloud study, participants evaluated the role model stories relatively positively, noting that they contributed to the normalization of chlamydia as well as to increased self-efficacy, skills, and intentions to perform preventive behaviors (STI testing, condom use, partner notification). However, some participants found the stories less relevant than the more informative texts about chlamydia present on Sense.info. Additionally, a few participants struggled to identify with the protagonists or questioned the authenticity of the uniformly positive outcomes depicted in the stories. The results will be discussed in more detail below.

When we began developing the personal stories, our primary objective was to strike a balance between the collected personal experiences of young people, which served as input for the stories, and the conditions for effectiveness of the BCP modeling. During the interviews conducted to shape the personal stories, we observed a strong alignment between the personal experiences and these conditions. For instance, the young individuals shared stories in which they initially faced challenges with chlamydia but later discovered the relative ease and positive outcomes of getting tested. This alignment was consistent with the condition that the model be a coping model rather than a mastery model, as well as with the reinforcement condition.^35^

However, in the think-aloud study, it was interesting to observe that some participants expressed skepticism about the authenticity of the personal stories due to the positive outcomes. Here, tension arose between the input from interviewees and wanting to meet theoretical requirements on the one hand, and the needs and wishes of other members of the target group on the other. While we strive to adhere to the conditions for effectiveness in the future and avoid unnecessarily portraying chlamydia as more serious than necessary given the current scientific discussion regarding the limited impact of chlamydia,^50^ we also recognize the potential value of placing a stronger emphasis on the initial challenges individuals faced and on potential negative side-effects of the target behavior. This might result in stories that are perceived as more authentic and can better assist people if they encounter difficult situations themselves.

In addition to the skepticism regarding the authenticity of the stories, some participants expressed an inability to identify with the protagonists and did not perceive the stories as (medically) relevant. This may result from the fact that participants in the think-aloud study were not required to have personal experience with chlamydia or be dealing with it at the time of the study. We did not ask participants in the think-aloud study if they were dealing with (suspicions of) chlamydia at the time of (enrolling in) the study, nor did we ask for their reasons to visit the Sense.info website at the time they enrolled in the study. Not acknowledging the stories as medically relevant might however also reflect a personal preference for more informational texts instead of narrative-based texts. The scoping review by Dudley et al. identified that combining narratives and informational messages is more effective than either strategy alone.^51,52^ To some extent, this is already the approach that Sense.info takes by offering both informative texts and narrative texts. To optimize this further, it might be beneficial to emphasize more strongly in the stories themselves that the personal stories contain medically relevant and accurate information that can assist readers in their own personal circumstances.

The lack of identification of the participants in the think-aloud study with the protagonists in the stories could also be attributed to the observation that some participants in the think-aloud study had less sexual experience than the protagonists of the stories. The most prominent similarity between the recipient and protagonist would then be age and/or gender. While such demographic variables may be important,^32^ they may not be sufficient on their own to create a strong sense of identification.^53,54^ Another factor contributing to identification could be, for example, the cultural background of the protagonists, which we did not ask them about and which is therefore not mentioned in the stories. This underscores the significance of developing role model stories with diverse protagonists from various backgrounds and experiences, allowing a broader audience to identify with one or more of the protagonists.

Despite the room for optimization, the results also indicated several positive outcomes. There was a positive impact on the normalization of chlamydia and the intention to engage in preventive behaviors. Additionally, participants reported an increase in self-efficacy and skills in accordance with modeling and SCT.^34,35,37,55^ Specifically regarding STI testing, the personal stories positively influenced participants’ confidence in their ability to get tested, and moreover, participants noticed and valued the inclusion of step-by-step plans within the stories, which helped enhance their skills in taking appropriate actions. However, normalization of chlamydia may not necessarily lead to prevention of chlamydia. Some studies have shown positive effects of sexual health interventions on self-efficacy and knowledge, with participants reporting that the intervention normalized having chlamydia, but biological data showed that more participants who received the intervention had an STI as a result of the mechanism that a reduction in felt stigma led to the intervention group to have more partners than the control group and therefore more STIs.^56,57^ Future research needs to assess whether a similar mechanism can be found in the context of our personal stories.

The analysis of actual use revealed that all stories had been accessed by Sense.info visitors. Page views, average time spent on the pages, and bounce and exit rates vary somewhat per story. To our knowledge, no concrete benchmarks regarding metrics such as acceptable bounce rates, exit rates, and other metrices exist for web-based public health interventions, as illustrated by several studies that have not reported specific standards yet merely compared metrics of different pages to gain insights into these metrics.^58^ In line with others, we believe that these metrics should always be analyzed in relation to the main outcomes a specific intervention has.^59^ For example, in the initial evaluation of Sense.info, we formulated assumptions on the potential impact of the chlamydia page considering the different motivations why visitors interact with the intervention (e.g., for informing themselves, for self-diagnosis reasons) and the several behaviors they could execute after this interaction (searching for more information and an STI test by following the link to the STI testing page, or by calling their own GP), that we then further studied in the exploration phase using a think-aloud study combined with semistructured interviews.^28,29^ A potential future strategy to gather more insightful usage data on the impact of personal stories would involve incorporating more links on the pages. For instance, stories that motivate readers to undergo an STI test at the Sense consultation hour could include a direct appointment booking link, allowing readers to schedule an appointment immediately. Similarly, stories promoting condom use could include a link to a page with addresses of places where condoms can be purchased. This approach would facilitate convenient access to relevant resources aligned with the message conveyed in the personal stories and would provide more insights into whether the main outcomes of the personal stories are achieved, adding to the qualitative findings. Potentially, this approach would also contribute to a higher perceived relevance of the stories and maybe to even more perceived self-efficacy and skills: this way, the initial steps for reaching the target behavior would be just a click away.

## Strengths and limitations

The CEP facilitated a structured and comprehensive assessment of the potential impact of the Sense.info chlamydia page. The mixed methods approach, incorporating a theoretical analysis of intended use, analysis of actual use through web analytics, and qualitative exploration of end-user perspectives, provided valuable insights into the practical relevance of applying behavior change theory in an intervention, and aligns with the growing trend of evaluating eHealth interventions using methods other than RCTs.^5–7^ Despite the meaningful results that were obtained, the CEP does not allow for claims about causality. A recent review highlighted various methods developed to address the limitations of the RCT while enabling statements about causality.^60^ However, most methods still require a confirming phase with an RCT or require selecting a primary outcome and studying specific intervention elements in separate procedures. This misaligns with interventions such as Sense.info, which have multiple outcomes that are targeted on one page. Therefore, the review concludes that the methods developed so far are not alternative but rather complementary to the RCT, and moreover, it asserts in line with the iterative approach of the CEP and with recent intervention evaluation criteria such as those from the UK Medical Research Council^5^ and the Dutch Partnership for Recognition of Interventions^7^ that evaluation should be considered throughout development, pilot testing, evaluation, and posttesting as a continuous cycle. Given the complexity of interventions, such as Sense.info, it is not sufficient anymore to rely on one method for garnering evidence. The CEP can be a valuable addition to other complementary methods to the RCT, especially given its focus on evaluation as well as optimization.

Related to the abovementioned lack of causal inferences with the CEP is that this study did not assess the real-life impact of the role model stories. An approach in which actual preventive and testing behavior would be assessed in the following months would be an interesting addition to this study. Moreover, variation in the stories remains important, for example in terms of the cultural background of the protagonists, so that as many as possible people are able to identify with the stories. Related to limited identification might be that participants did not necessarily have to have experienced chlamydia at the time of the study, which might render the topic of chlamydia less relevant and might have been a barrier to identification. Finally, most of the STI test results in the stories were positive, whereas the target behavior was testing rather than testing positive. However, it should be noted that in the Dutch context, a new policy has recently been introduced where people are only tested for chlamydia if they have symptoms.^50,61^ In this sense, we argue that the focus on positive tests in the stories is justifiable.

## Conclusions

This study has given us insight into the potential impact of role model stories developed in collaboration with young people for sexual health promotion and insights for future optimization of such stories. Additionally, this study has provided us with insights into the use of the CEP for evaluating and optimizing web-based public health interventions. It guided us in understanding how to enhance these stories in future optimization rounds and align them more closely with the needs of other members of the Sense.info group of interest. Given our findings and the current trend toward more qualitative and mixed methods evaluation of web-based interventions, we recommend using the CEP for evaluating and optimizing web-based public health interventions.

## Supplemental Material

sj-docx-1-dhj-10.1177_20552076241308447 - Supplemental material for Systematic optimization and evaluation of a Dutch sexual health intervention: Role model stories for chlamydia prevention, testing, and treatment Supplemental material, sj-docx-1-dhj-10.1177_20552076241308447 for Systematic optimization and evaluation of a Dutch sexual health intervention: Role model stories for chlamydia prevention, testing, and treatment by Gido Metz, Rosa RLC Thielmann, Hanneke Roosjen, Sarah E. Stutterheim and Rik Crutzen in DIGITAL HEALTH

sj-docx-2-dhj-10.1177_20552076241308447 - Supplemental material for Systematic optimization and evaluation of a Dutch sexual health intervention: Role model stories for chlamydia prevention, testing, and treatment Supplemental material, sj-docx-2-dhj-10.1177_20552076241308447 for Systematic optimization and evaluation of a Dutch sexual health intervention: Role model stories for chlamydia prevention, testing, and treatment by Gido Metz, Rosa RLC Thielmann, Hanneke Roosjen, Sarah E. Stutterheim and Rik Crutzen in DIGITAL HEALTH

sj-docx-3-dhj-10.1177_20552076241308447 - Supplemental material for Systematic optimization and evaluation of a Dutch sexual health intervention: Role model stories for chlamydia prevention, testing, and treatment Supplemental material, sj-docx-3-dhj-10.1177_20552076241308447 for Systematic optimization and evaluation of a Dutch sexual health intervention: Role model stories for chlamydia prevention, testing, and treatment by Gido Metz, Rosa RLC Thielmann, Hanneke Roosjen, Sarah E. Stutterheim and Rik Crutzen in DIGITAL HEALTH
