# Supplementary material for: Systematic optimization and evaluation of a Dutch sexual health intervention: Role model stories for chlamydia prevention, testing, and treatment
Source: Digit Health. 2025 Jan 23;11:20552076241308447. doi: 10.1177/20552076241308447 (PMC11755532; doi:10.1177/20552076241308447)
Supplement: sj-docx-1-dhj-10.1177_20552076241308447 - Supplemental material for Systematic optimization and evaluation of a Dutch sexual health intervention: Role model stories for chlamydia prevention, testing, and treatment [file sj-docx-1-dhj-10.1177_20552076241308447.docx]

**Appendix A**

**Questions/Checklist for interviews about personal experiences with chlamydia**

*Suspicions about chlamydia*

1. Why did you suspect you had chlamydia?
   1. Complaints/symptoms of chlamydia
   2. Notified about chlamydia by sex partner(s)
   3. Viewed information about chlamydia on Sense.info
   4. Viewed information/heard about chlamydia in a place other than Sense.info, namely…
   5. Otherwise, namely…
2. How did you feel when you suspected you had chlamydia?
3. If you were warned by sex partner(s): how?
   1. Via a ‘partnerwaarschuwing [‘partner notification’, official application used by health care providers] message
   2. My sexual partner(s) told me personally
   3. Otherwise, namely….
4. If you were warned by sex partner(s): how did you feel when you heard it?

*Testing*

1. Have you been tested for chlamydia?
2. Did the test say you had chlamydia?
3. Did you have the test done at the GGD [sexual health center]/General Practitioner/Commercial provider?
4. Can you tell me something about your reasons for testing at the GGD/GP/commercial provider specifically (and therefore not at the other provider(s)?)
5. What motivated you to test (were you scared by the symptoms you had, did you want reassurance, etc.)?
6. What steps did you take to make a test appointment (call/online/…)?
7. How did you find it making an appointment (easy/difficult/awkward)?
8. What did you think of the conversation with the nurse/doctor during the STI test appointment?
9. What did you think of the (physical) examination during the STI test appointment?
10. How did you feel before and after the test?
11. Have your thoughts about chlamydia changed after the test/visit to the doctor/nurse? (‘at first I expected it to be very serious, but now I think…’)
12. If not tested: can you tell about your reasons for not getting tested?
13. Do you have any tips regarding testing?
14. Is there anything else you would like to say about testing?

*Treatment*

1. Have you been treated for chlamydia?
2. Did you know in advance what the treatment entails?
3. How did you find the treatment?
4. Do you have any tips regarding the treatment?
5. Is there anything else you would like to say about the treatment?

*Notify partner(s)*

1. Have you notified your sexual partner(s) about chlamydia?
2. How did you notify your sexual partner(s)?
   1. Myself, e.g., during a (telephone) conversation.
   2. Via ‘partnerwaarschuwing’ [‘partner notification’, official application used by health care providers]
   3. Otherwise, namely…
3. How did you feel about notifying your sexual partner(s)?
4. How did you approach the conversation?
5. How did you feel before and after the conversation with your sexual partner(s)?
6. Do you have any tips about notifying your sexual partner(s)
7. Is there anything else you would like to say about notifying your sexual partner(s)?

*After chlamydia*

1. Have things changed for you after having had chlamydia/ after having been notified about chlamydia/ (or more generally: after your experience with chlamydia)?
2. What tip or message would you like to give to young people like you?
3. Is there anything else you would like to tell us about your experience with chlamydia?
