# Supplementary material for: Systematic optimization and evaluation of a Dutch sexual health intervention: Role model stories for chlamydia prevention, testing, and treatment
Source: Digit Health. 2025 Jan 23;11:20552076241308447. doi: 10.1177/20552076241308447 (PMC11755532; doi:10.1177/20552076241308447)
Supplement: sj-docx-2-dhj-10.1177_20552076241308447 - Supplemental material for Systematic optimization and evaluation of a Dutch sexual health intervention: Role model stories for chlamydia prevention, testing, and treatment [file sj-docx-2-dhj-10.1177_20552076241308447.docx]

**TELEPHONE CONVERSATION IN ADVANCE**

- Brief explanation of research
- Which device do you usually use when visiting Sense.info? Phone, laptop, tablet?
- Then please use that device for the research
- Schedule an appointment (Or, if possible: we can do the session right away)
- You will receive a zoom link, information letter + link to Informed Consent form by email

**INTRODUCTION/EXPLANATION THINK ALOUD**

- Welcome
- Introduce myself
- Have the participant introduce themselves
- Do you have experience with research?
- Explanation of sense and research based on information letter
- We want to improve Sense.info. We are curious to hear what you think about Sense.
- Sometimes people find it uncomfortable to talk about sex. You can say anything here and there is no right or wrong. No one will know that you participated and what exactly you said.
- I don't work for Sense. I didn't create the website either. So you can say anything you like.
- We only ask you for your opinion about the site and nothing about your own experiences.
- You can stop at any time.
- I will record the screen and audio (not your face) so I can transcribe the interview later
- Sometimes I will write something down. That's so I can ask another question about it later.
- It is not a quick search competition. You can look around at your leisure.
- You can view everything and click on everything you want.
- Browse the site as you would if I were not here.
- Questions? Everything clear?
- Silent for more than 10 seconds? Cue “think out loud.”
- Afterwards I will ask you to email me your house address. This is so that I can justify to the university's finance department who I gave the tourist voucher to. This data will not be used for the research.

**SIGN INFORMED CONSENT FORM**

[Link]

**PRACTICE ROUND**

- Are you in a place where you won’t be disturbed?
- Open a new browser window
- Share your screen
- Assignment: go to Trimbos.nl, browse the homepage and think out loud.
- Find the phone number of the help line while thinking out loud.

**START RESEARCH**

- All other tabs closed?
- Open a new browser window
- Share your screen
- **START RECORDING**
- Assignment: go to Sense.info and look around the homepage. In the meantime, think out loud.
- Assignment: now find the page about chlamydia, view the information on this page as you would if I were not here, and think out loud in the meantime.

**SEMI-STRUCTURED INTERVIEW**

**SEARCH**

- I asked you to find the chlamydia page and look at that information. How did you find that looking up?
- Do things need to be adjusted to make this page easier to find?

**CHLAMYDIA**

- Have you seen this page before? With what purpose?

**Determinants**

*Attitudes, Knowledge, Trust, Risk Perception (Susceptibility & Severity), Self-Efficacy, Response Efficacy*

- We're curious if your ideas about chlamydia have changed after looking at this information. Can you tell us something about that?

**Probes:**

- Trust
  - How reliable do you find the information on this page?
- Risk perception
  - Susceptibility: Can you tell me about whether your ideas about how quickly you can contract chlamydia have changed after looking at this page?
  - Severity: Can you tell me whether your ideas about the consequences of chlamydia have changed after viewing this page?
- Safe sex
  - Attitudes: Can you tell me something about whether your ideas about safe sex have changed after looking at this page?
  - Knowledge: To what extent have you learned new things about safe sex after viewing this page?
  - Self-efficacy: Do you think you can have safe sex? / After viewing this page, to what extent are you confident that you will succeed in having safe sex?
  - Response efficacy: After viewing this page, to what extent do you think that safe sex helps prevent chlamydia in others?
  - Elements/page: Since we're talking specifically about safe sex, can you tell me what you think about the elements on this page?
- STI test
  - Attitudes: Can you tell us if your ideas about an STI test have changed after viewing this page?
  - Knowledge: To what extent have you learned new things about an STI test after viewing this page? And about where you can take an STI test?
  - Self-efficacy: Do you think you can do that, an STI test? / After viewing this page, to what extent are you confident that you will be able to take an STI test?
  - Response efficacy: After viewing this page, to what extent do you think testing yourself helps prevent chlamydia in others?
  - Elements/page: Since we're talking specifically about STI testing, can you tell me what you think about the elements on this page?
- Treatment of chlamydia
  - Attitudes: Can you tell us if your ideas about chlamydia treatment have changed after looking at this page?
  - Knowledge: To what extent have you learned new things about the treatment after viewing this page?
  - Self-efficacy: Do you think you can get yourself treated against chlamydia? / To what extent are you now confident that you will be able to get treated against chlamydia?
  - Response efficacy: To what extent do you think that treatment helps to prevent chlamydia in others?
  - Elements/Page: Since we're talking specifically about chlamydia treatment, can you tell me what you think about the elements on this page?
- Notify sexual partners
  - o Attitudes: Can you tell us if your ideas about telling your sex partners that you have chlamydia have changed after viewing this page?
  - o Knowledge: After viewing this page, to what extent have you learned new things about telling sexual partners that you have chlamydia?
  - o Self-efficacy: Do you think you can tell your sex partner? / To what extent are you confident that you will be able to warn your sex partner?
  - o Response efficacy: To what extent do you think that telling your sex partner helps prevent chlamydia in others?
  - o Elements/page: Now that we're talking specifically about sex partner, can you tell me what you think about the elements on this page?

**Optimized or new elements:**

What did you think of:

- Personal stories
  - Identification: To what extent can you recognize/put yourself in the shoes of the young person in the story?
  - Goal/subbehaviors: To what extent would you do something after reading this story?/What would you do after reading this story?
  - Descriptive standard: To what extent does the person in the story motivate you to take an STD test yourself/get treated against chlamydia/warn your partner?
  - Skills: After reading this story, can you tell me what steps you need to take to have safe sex/take an STD test/get treatment for chlamydia/warn your partner?
  - Self-efficacy: After reading this story, to what extent are you confident that you would be able to have safe sex/take an STD test/get treatment for chlamydia/warn your partner?
- Date 'last edited on...by infectious disease control doctor'
- Link to STI test (higher on the page)
- Link to Advieschat
- Link to Soa Aids Nederland and Thuisarts

Optimized or new elements: if not interacted with:

- You didn't click on the Personal Stories, can you tell us something about your reasons for that?
- You didn't click on the Advice Chat, can you tell us something about your reasons for that?
- You didn't click on STD test, can you tell us something about your reasons for that?

**To conclude**

- Anything else you want to add to what you just told me?

**DEBRIEFING**

- Answer any questions from participant

- If necessary, refer to Sense/Soa Aids Nederland, in case of questions about sexual health, etc.
